# Supplementary material for: SUMOylation of SAMHD1 at Lysine 595 is required for HIV-1 restriction in non-cycling cells
Source: Nat Commun. 2021 Jul 28;12:4582. doi: 10.1038/s41467-021-24802-5 (PMC8319325; doi:10.1038/s41467-021-24802-5)
Supplement: Supplementary file 1 — Supplementary information [file 41467_2021_24802_MOESM1_ESM.pdf]

Supplementary Information

SUMOylation of SAMHD1 at Lysine 595 is required for HIV-1 restriction in non-cycling cells

Martinat et al.

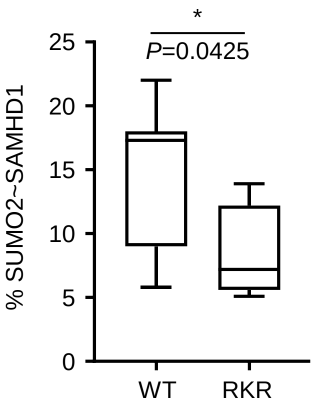

Supplementary Figure 1: Quantification of the SUMOylated fraction of SAMHD1 (related to Fig. 1a). Box and whisker plots showing the ratio between mono-SUMOylated and unmodified SAMHD1 variants in transfected HEK 293T cells. Quantification was performed by densitometric analysis with FiJi-Image J (n = 13 WT, n = 5 RKR). Box plots extend from the 25th to 75th percentiles, the middle line is the median and whiskers go down to the smallest and up to the largest value. Statistical significance was determined using a paired, two-tailed Student's t-test (*: $p < 0.05$).

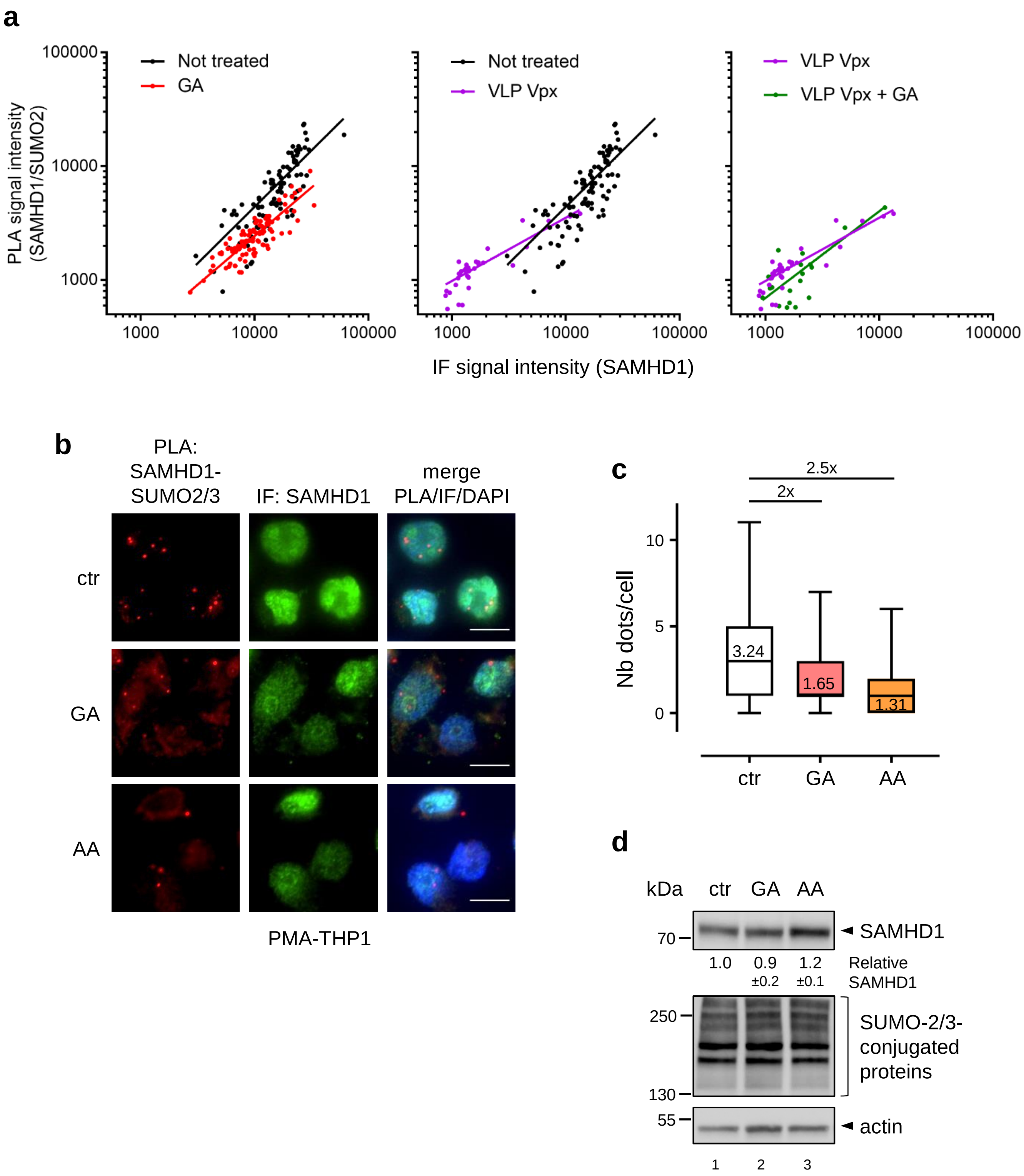
a
Not treated
GA
VLP Vpx
VLP Vpx + GA
100000
10000
1000
PLA signal intensity
(SAMHD1/SUMO2)
1000
10000
100000
IF signal intensity (SAMHD1)
b
PLA:
SAMHD1-
SUMO2/3
IF: SAMHD1
merge
PLA/IF/DAPI
ctr
GA
AA
PMA-THP1
c
2.5x
2x
10
5
0
Nb dots/cell
3.24
1.65
1.31
ctr
GA
AA
d
kDa
ctr
GA
AA
70
SAMHD1
1.0
0.9
±0.2
1.2
±0.1
Relative
SAMHD1
250
130
SUMO-2/3-
conjugated
proteins
55
actin
1
2
3

Supplementary Figure 2. SUMOylation inhibitors reduce the interaction between SUMO2/3 and SAMHD1 without affecting its expression levels (related to Figs. 1d, 1e and 6). a. Quantitative analysis of the PLA and IF signals was performed with the Icy software for isolated MDMs: NT (95), GA (105), VLP-Vpx (35), VLP-Vpx+GA (25). **b.** Differentiated THP-1 cells were incubated with ginkgolic acid (GA, 50µM), anacardic acid (AA, 50µM) or DMSO (ctr) for 2 hours. Cells were stained with mouse anti-SAMHD1 (Ab67820) and rabbit anti-SUMO2/3 (Ab3742) antibodies and processed for PLA as in Fig. 1d. Scale bar = 10 µm. Representative images are shown (n=2). **c.** The SAMHD1-SUMO2/3 PLA signal was quantified manually for n=150 cells with the same thresholding parameters across parallel samples using the Icy software. Box and whisker plots show the number of dots per cell in one representative experiment (n=2). Boxes extend from the 25th to 75th percentiles, the middle line is the median and whiskers go down to the smallest and up to the largest value. Mean values are indicated in the boxes. **d.** Proteins (20 µg total proteins/line) contained in the crude lysate of differentiated THP1 treated with GA or AA (50µM, 2h) or DMSO (ctr) were separated by migration on a 4-15% SDS-PAGE gel and, next, visualized by immunoblotting using antibodies against SAMHD1 (Ab67820), SUMO2/3 (Ab3742), or actin. The intensity of bands corresponding to SAMHD1 and actin, used as loading control, was determined by densitometry with ImageJ software. The SAMHD1/actin ratio in DMSO-treated cells was set to 1. Images from one representative experiment are shown, while the quantification data represent the mean ± SD of all experiments (n=3).

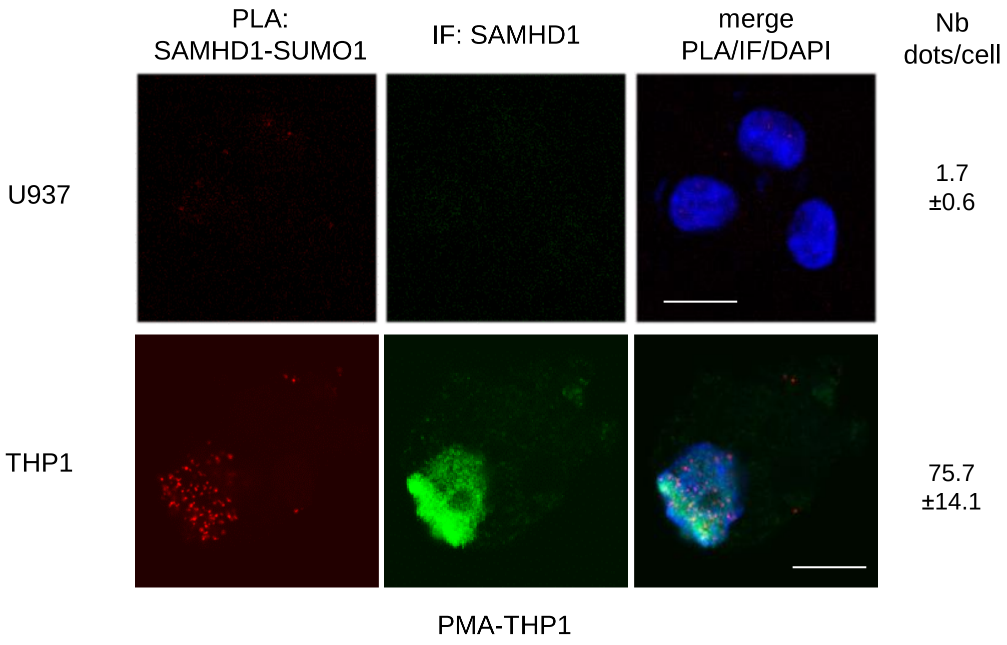

Supplementary Figure 3. SAMHD1 interacts with SUMO1 in differentiated THP1, but not U937 cells (related to Fig. 1d). Differentiated THP1 or U937 cells were co-stained with mouse anti-SAMHD1 (Ab67820) and rabbit anti-SUMO1 (Ab32058) antibodies and treated as in figure 1d. Data from one representative experiment are shown (n=2). The SAMHD1-SUMO1 PLA signal (mean ± SD) was quantified for 10 cells as in supplementary Fig. 1c. Scale bar = 10 µm.

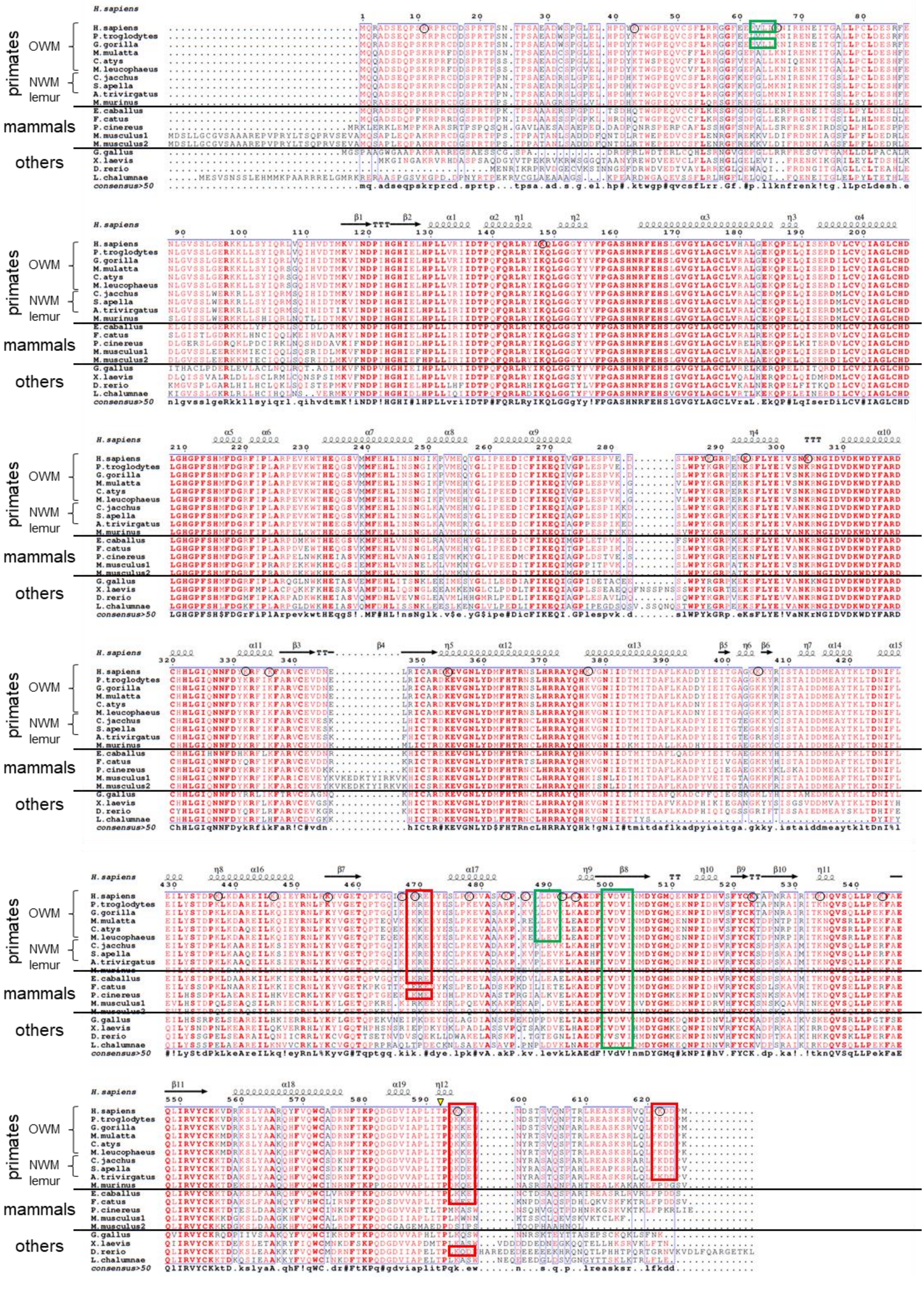

Supplementary Figure 4. Multi-alignment of SAMHD1 sequences from different species (related to Figs. 2 and 4). Sequences of SAMHD1 isoforms from various vertebrate species were aligned using the MultiAlin software[1] and visualized using ESPRIT[2]. Lysine (K) residues are highlighted in black circles. SUMOylation sites centered on K469, K595 and K622 are boxed in red, while SIM1, SIM2 and SIM3 are boxed in green. The black arrowhead points at the phosphorylatable T592 residue.

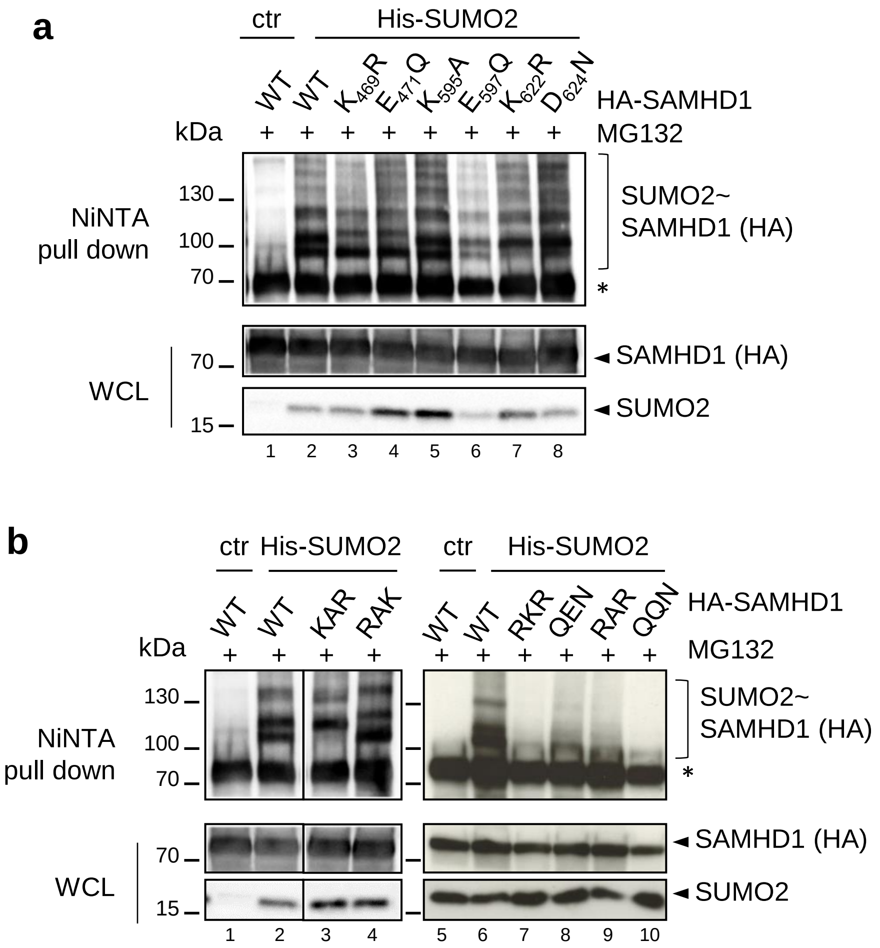

Supplementary Figure 5. Residues K469 and K622 are modified by SUMO chains that accumulate upon proteasome inhibition (related to Fig. 2). a. HEK 293T cells were transfected with plasmids encoding HA-SAMHD1 WT or single or **b.** multiple SUMO-site mutants, Ubc9 and His-SUMO2 or the control empty plasmid (ctr). After 24 hours, samples were treated MG132 (3µM, ON) and then were processed as in Fig. 1a. K469, K595 and/or K622 were mutated into R or A, while E471, E597 and/or D624 were mutated to either Q or N as described in Fig. 2c. Lanes 1 to 4 in panel b are derived from the same blot but were not adjacently loaded. WCL: whole cell lysate. *, nonspecific binding of unmodified SAMHD1 on Ni-NTA beads. Results of one representative experiment are shown (n ≥ 2).

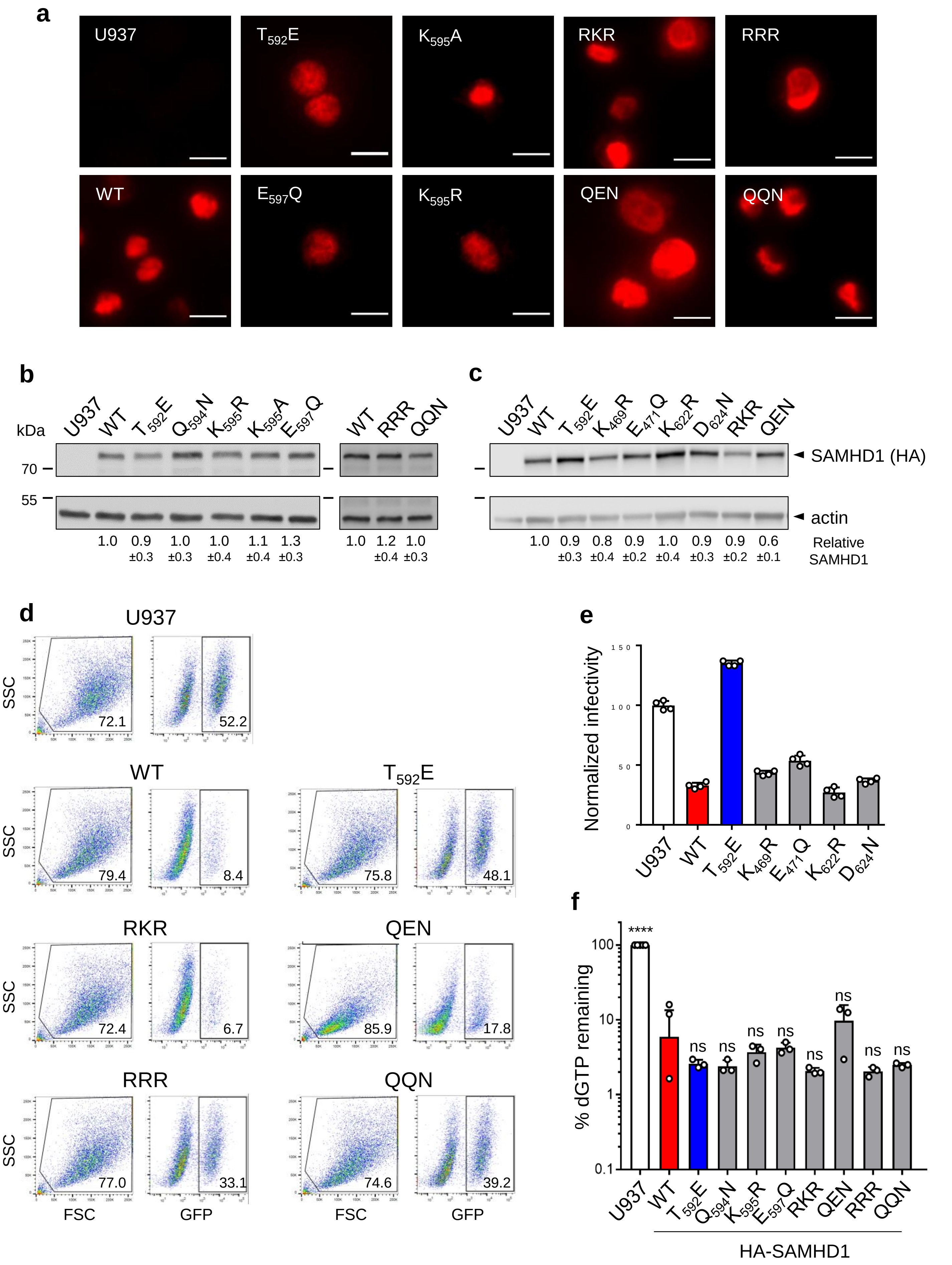
a
U937
T592E
K595A
RKR
RRR
WT
E597Q
K595R
QEN
QQN
b
kDa
U937 WT T592E Q594N K595R K595A E597Q WT RRR QQN
70
55
1.0 0.9 ±0.3 1.0 ±0.3 1.0 ±0.4 1.1 ±0.4 1.3 ±0.3 1.0 1.2 ±0.4 1.0 ±0.3
c
U937 WT T592E K469R E471Q K622R D624N RKR QEN
SAMHD1 (HA)
actin
1.0 0.9 ±0.3 0.8 ±0.4 0.9 ±0.2 1.0 ±0.4 0.9 ±0.3 0.9 ±0.2 0.6 ±0.1
Relative SAMHD1
d
U937
SSC
72.1
52.2
WT
79.4
8.4
T592E
75.8
48.1
RKR
72.4
6.7
QEN
85.9
17.8
RRR
77.0
33.1
QQN
74.6
39.2
FSC
GFP
e
Normalized infectivity
150
100
50
0
U937 WT T592E K469R E471Q K622R D624N
f
% dGTP remaining
100
10
1
0.1
****
ns
U937 WT T592E Q594N K595R E597Q RKR QEN RRR QQN
HA-SAMHD1

Supplementary Figure 6. Characterization of SUMOylation-defective SAMHD1 mutants (related to Fig. 3). a. The localization of SAMHD1 mutants was assessed in differentiated U937 cells by immunofluorescence using an anti-HA antibody followed by anti-isotype secondary antibody coupled to the $Alexa_{594}$ dye. Images were acquired with an Axiovert 200 M inverted microscope, using a Plan Apo 63/1.4-N oil immersion objective. Images from one representative experiment are shown (n = 2). Scale bar = 10 µm. **b.** The expression levels of SAMHD1 single or **c.** multiple SUMO-site variants stably expressed in U937 cells were monitored after PMA treatment in the total cell extract by immunoblotting (15 µg total proteins/lane). Band intensities were quantified by densitometry with ImageJ software. The WT SAMHD1/actin ratio was set to 1. One representative immunoblot image is shown (n = 11 WT and 7 $T_{592}E$, 6 $E_{471}Q$ and QEN, 5 $K_{496}R$ and $K_{622}R$, 4 $D_{624}N$ and RKR), while the quantification data represent the mean ± SD of all the experiments. **d.** Gating strategy to quantify HIV-infected cells. Differentiated U937 cell lines challenged VSVg/HIV-1ΔEnvGFP virus were fixed and then analyzed by flow cytometry. Live cells were gated (SSC/FSC panels) and the proportion of GFP-positive cells was quantified (SCC/GFP panels). One sample per cell line corresponding to the experiment shown in Fig. 3b are presented as example. **e.** U937 cell lines expressing the indicated HA-SAMHD1 variants (2 independently generated cell lines) were infected with VSVg/HIV-1ΔEnvGFP virus and analyzed as in Fig. 3b. Data (mean ± SD) from one representative experiment performed in 4 technical replicates are shown (n = 3). **f.** The cellular dGTP levels of differentiated U937 cell lines expressing SAMHD1 variants were quantified as in Fig. 3d. The dGTP levels (%) of SAMHD1-expressing cells were calculated relative to those of parental U937 set to 100. Bars show the mean ± SD (n = 3). Statistical significance was assessed by one-way ANOVA with Dunnett's multiple comparison post-test. ****: $p<0.0001$. ns: not significant, $p>0.05$.

Results for putatifs SIM [PSmax=38.183 | Cut-off=0.269]					
Position site	Sequence	Type	α/S stretch	PS	DB Hit
AA 62-65	LRRGGFEEPVLLKNIRENEI	SIM Type 4	[N][SIM][N]	0.382	
AA 488-491	VASAKPKVLLDVKLKAEDFI	SIM Type 1	[N][SIM][N]	0.335	1
AA 499-502	VKLKAEDFIVDVINMDYGMQ	SIM Type β	[N][SIM][N]	4.191	1
AA 500-503	KLKAEDFIVDVINMDYGMQE	SIM Type α	[N][SIM][N]	0.665	

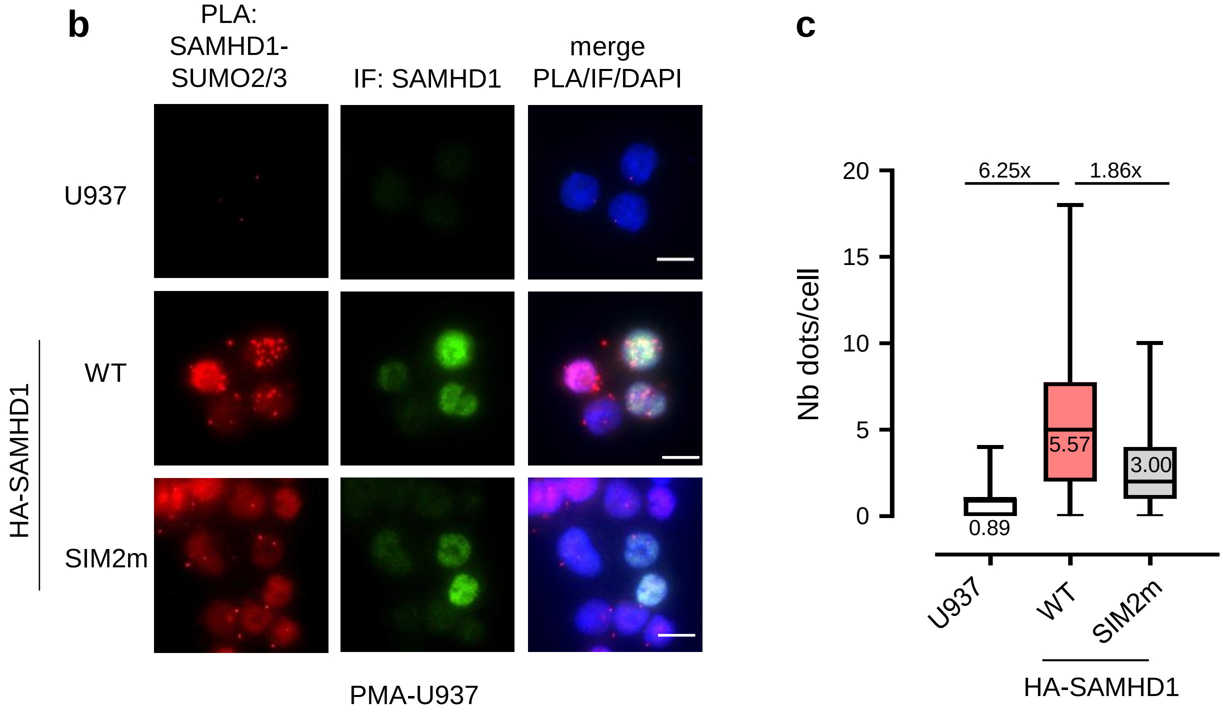

Supplementary Figure 7. The SIM2 of human SAMHD1 is important for the non-covalent interaction with SUMO proteins (related to Fig. 4). a. *In silico* analysis with JASSA[3] predicts the presence of several SIMs in the sequence of human SAMHD1. **b.** Differentiated U937 cell lines stably expressing WT or SIM2m SAMHD1 variants were processed for PLA as in Fig. 1d. Parental U937 cells were used as negative control. Scale bar = 10 µM. Images from one representative experiment are shown (n = 2). **c.** The SAMHD1-SUMO2/3 PLA signal of differentiated U937 cell lines [WT (56), SIM2m (36)] was quantified as in Supplementary Fig. 2c. Box plots, which extend from the 25th to 75th percentiles with the middle line representing the median and whiskers going down to the smallest and up to the largest value, show the number of dots per cell in one representative experiment (n = 2). Mean values are indicated.

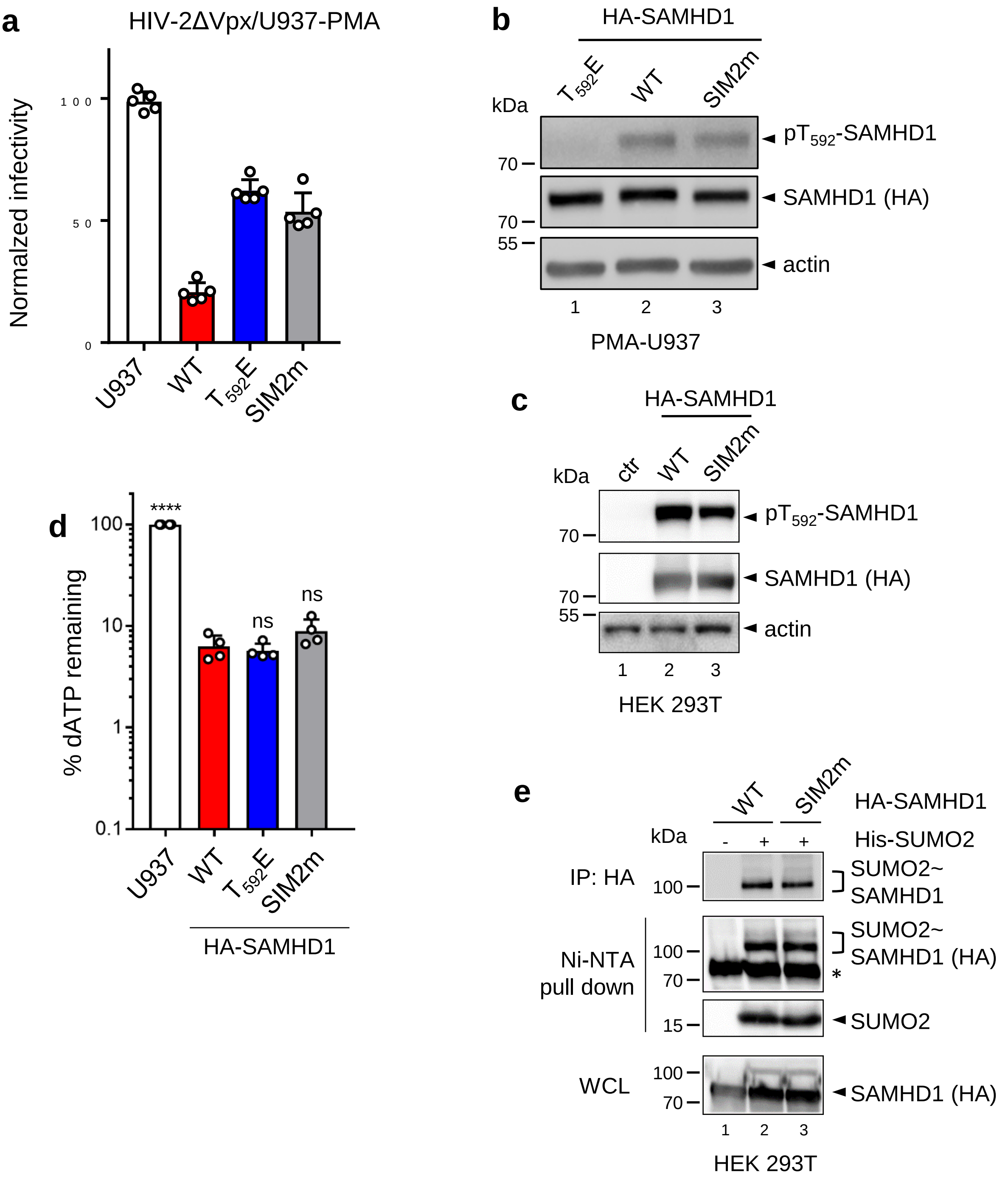

Supplementary Figure 8. Functional characterization of SAMHD1 SIM2m variant (related to Fig. 4). a. U937 cells stably expressing HA-SAMHD1 WT or mutants (2 independent transductions) were differentiated by PMA treatment and then challenged with VSVg/HIV-2ΔVpx expressing GFP as reporter. Analysis was performed as in Fig. 3b. Bars represent the mean ± SD of 4 technical replicates. Data from one representative experiment are shown (n=2). **b.** The levels of total and phosphorylated SAMHD1 were monitored in the crude extract of differentiated U937 cell lines or **c.** transfected HEK 293T cells using an anti-HA or anti-pT592 specific antibody (10 µg total proteins/line). Actin was used as loading control. **d.** The levels of dATP were quantified and normalized as in Fig. 3d. The dNTP levels of U937 were set to 100%. Bars show the mean ± SD (n=4). Statistical significance was assessed by one-way ANOVA test. ****: $p<0.0001$. ns: not significant, $p>0.5$. **e.** HEK 293T cells overexpressing WT or SIM2m HA-SAMHD1 mutants, Ubc9 and His-SUMO2 were treated as in Fig. 4f. Results of one representative experiment are shown (n=2). *, nonspecific binding of unmodified SAMHD1 on Ni-NTA beads.

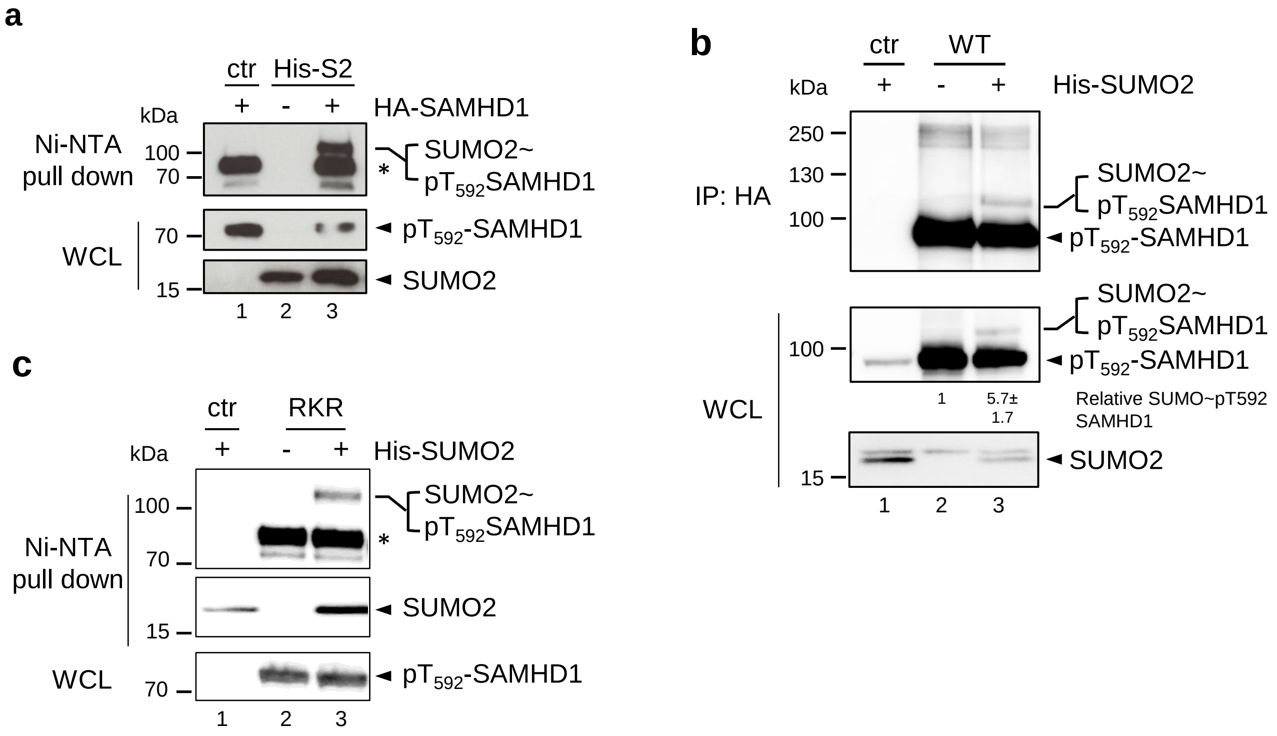

Supplementary Figure 9. A fraction of SAMHD1 is co-modified by T592 phosphorylation and K595 SUMOylation (related to Fig. 5). a. Lysates of HEK 293T cells overexpressing HA-SAMHD1 and/or His-SUMO2 were incubated with Ni-NTA beads **b.** or HA-matrix beads. Proteins from the input and the eluates were separated by migration on a 4-15% SDS-PAGE gel and, next, visualized by immunoblotting using antibodies specifically recognizing the T592 phosphorylated SAMHD1 form or SUMO2/3. Immunoblot image is representative of n =2 independent co-precipitation experiments. Densitometric quantification of band intensities with Image J was used to define the fraction of SUMOylated SAMHD1 relative to the T592 phosphorylated specie in n=4 independent experiments (mean ± SD). WCL: whole cell lysate. **c.** HEK 293T cells overexpressing HA-SAMHD1 RKR mutant and/or His-SUMO2 were processed for Ni-NTA pull down as described in Fig. 1a. Immunoblotting was preformed using anti-pT592 SAMHD1 or anti SUMO2/3 antibodies. *, nonspecific binding of non-SUMOylated pT592~SAMHD1 forms on Ni-NTA beads.

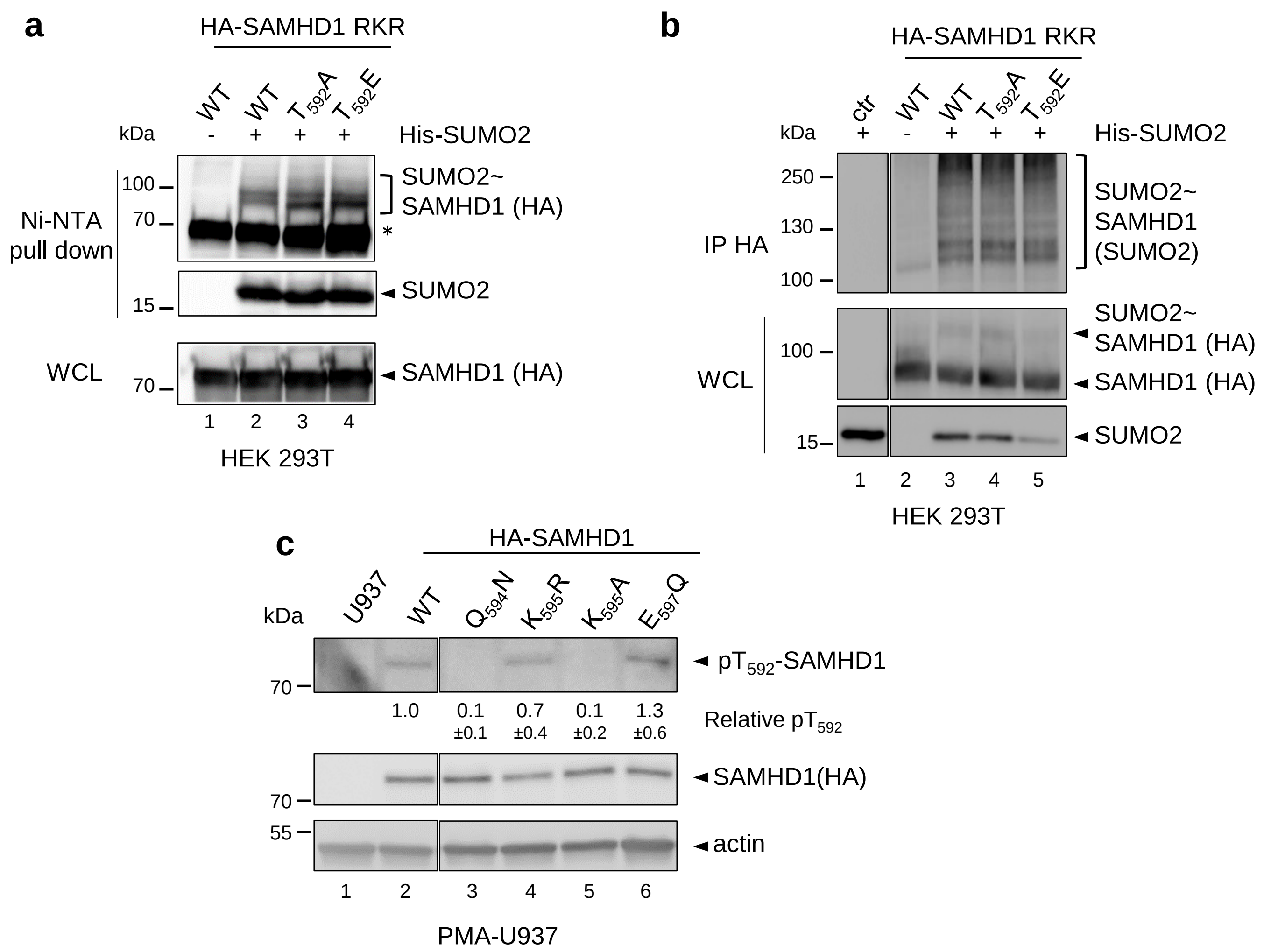

Supplementary Figure 10. The phosphorylation status of T592 does not influence K595 SUMOylation, while SUMOylation-deficient K595A SAMHD1 mutant is hypophosphorylated (related to Fig. 5). a. HEK 293T cells overexpressing the indicated HA-SAMHD1 variants, Ubc9 and His-SUMO2 were split in two equal aliquots and processed for Ni-NTA pull down or **b.** IP was performed as described in Fig 4F. WCL: whole cell lysate. Image from one representative experiment is shown (n = 4). **c.** The expression levels of SAMHD1 variants stably expressed in differentiated U937 cells were monitored in the total cell extract by immunoblotting (20 µg total proteins/lane). Band intensities were quantified as in Fig. 5a. The pT592/total SAMHD1 ratio for WT SAMHD1-expressing cells was set to 1.One representative image is shown (n = 3), while quantitative date are the mean ± SD of all the experiments. Lanes in panels b and c are derived from the same blot but were not adjacently loaded.

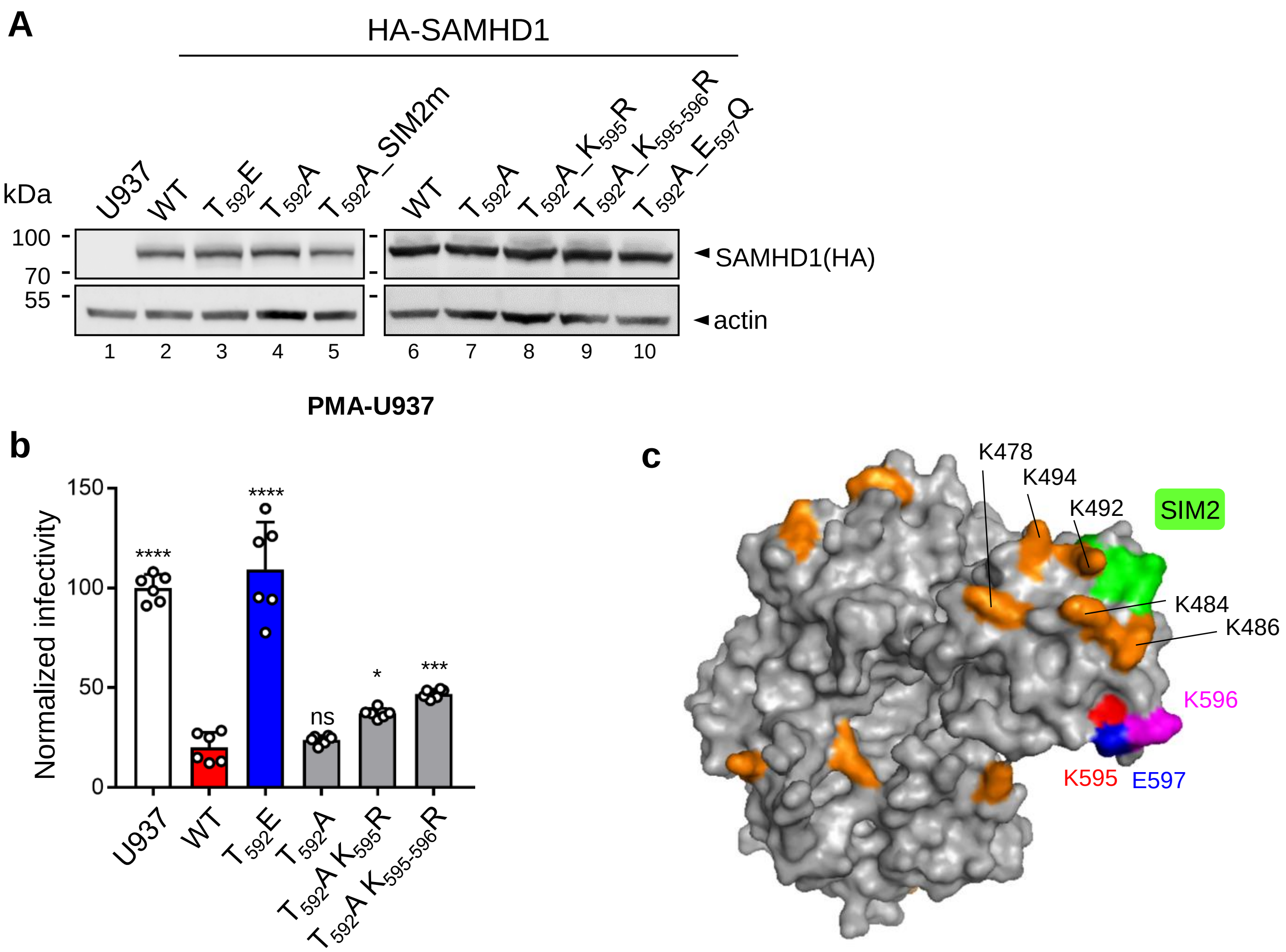

Supplementary Figure 11. Alanine substitution of K596 has minor impact on the antiviral activity of SAMHD1 T592A-K595R variant, but many SUMOylatable K residues lie near SIM2 (related to Fig. 5). **a.** The levels of total and phosphorylated SAMHD1 were monitored in the crude extract of differentiated U937 cell lines using an anti-HA or anti-pT592 specific antibody (15 µg total proteins/lane). Actin was used as loading control. One representative image of two independent experiments is shown. **b.** U937 cells stably expressing HA-SAMHD1 WT or mutants (2 independent transductions) were differentiated by PMA treatment and then challenged with the VSVg/HIV-1*EGFP* virus. Analysis was performed as in Fig. 5c. Bars show the mean ± SD from one representative experiment performed in 3 technical replicates ($n \geq 2$). Statistical significance was assessed by one-way ANOVA test. *: $p<0.05$; ***: $p< 0.001$; ****: $p<0.0001$. ns: not significant. **c.** Position of SUMOylatable K residues (orange, unless otherwise specified) lying near SIM2 (green) and the SUMOylation site centered on K595 (red), which includes K596 (fuchsia) and E597 (blue), within one protomer of human SAMHD1 tetramer (PDB: 4BZC).

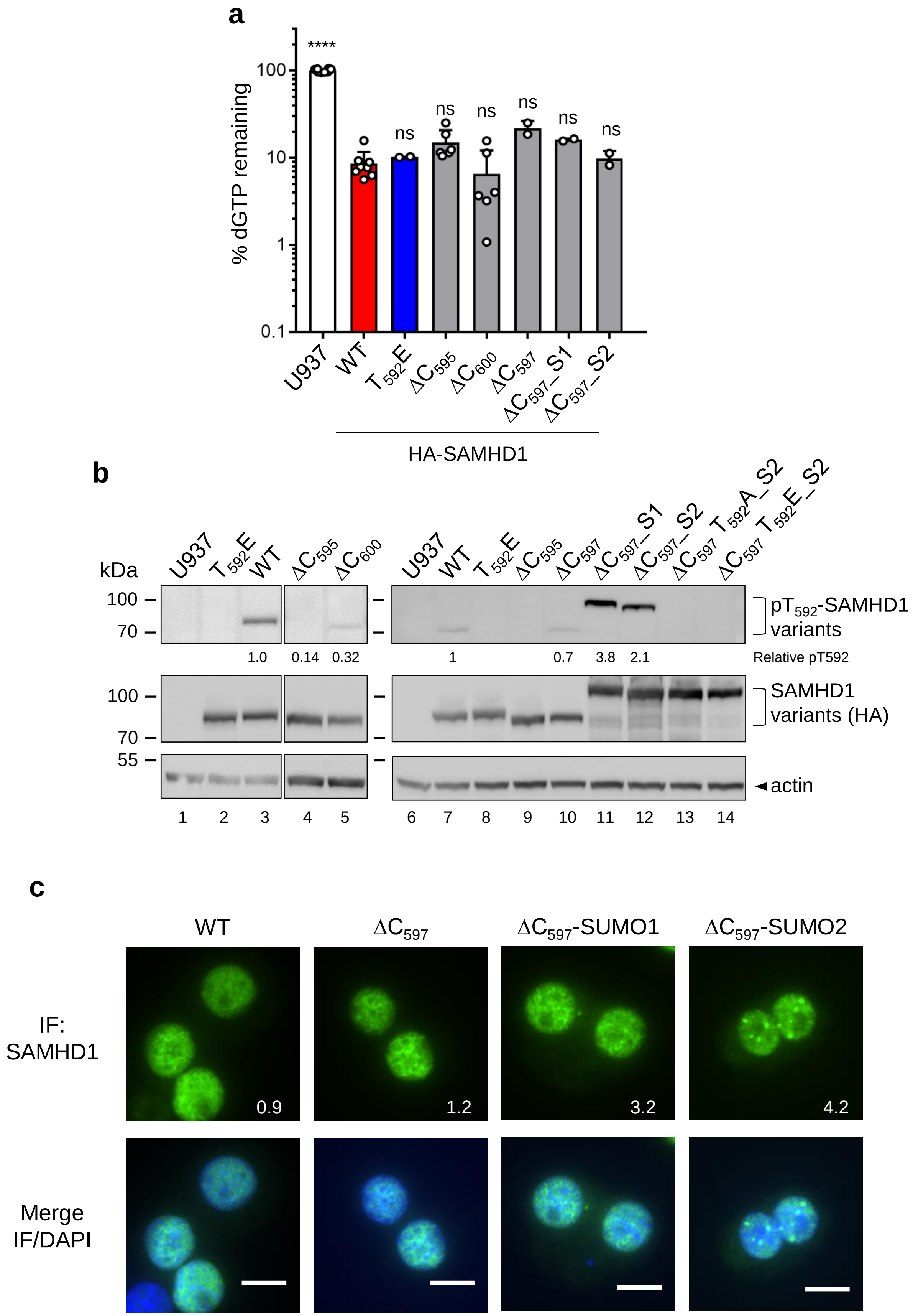
a
****
100
10
1
0.1
% dGTP remaining
ns
ns
ns
ns
ns
ns
U937
WT
T592E
ΔC595
ΔC600
ΔC597
ΔC597_S1
ΔC597_S2
HA-SAMHD1
b
kDa
U937
T592E
WT
ΔC595
ΔC600
U937
WT
T592E
ΔC595
ΔC597
ΔC597_S1
ΔC597_S2
ΔC597 T592A_S2
ΔC597 T592E_S2
100
70
pT592-SAMHD1 variants
1.0
0.14
0.32
1
0.7
3.8
2.1
Relative pT592
100
70
SAMHD1 variants (HA)
55
actin
1
2
3
4
5
6
7
8
9
10
11
12
13
14
c
WT
ΔC597
ΔC597-SUMO1
ΔC597-SUMO2
IF: SAMHD1
0.9
1.2
3.2
4.2
Merge IF/DAPI

Supplementary Figure 12. Characterization of SAMHD1 C-terminal truncation mutants and SUMO-fusions (related to Fig. 5). a. The dGTP levels of differentiated U937 expressing the indicated HA-SAMHD1 variants were quantified and normalized as in Fig. 3d. Bars show the mean ± SD ($n \geq 2$). Statistical significance was assessed by one-way ANOVA with Dunnett's multi-comparisons post-test. ****: $p<0.0001$. ns: not significant, $p>0.5$. **b.** The levels of total and phosphorylated SAMHD1 were monitored in the crude extract of differentiated U937 cell lines using an anti-HA or anti-pT592 specific antibody (15 µg total proteins/lane). Actin was used as loading control. Band intensities were quantified as in Fig. 5a. The pT592/total SAMHD1 ratio for WT SAMHD1-expressing cells was set to 1. Images from one representative experiment are shown (n = 2). Lanes 1 to 5 are derived from the same blot but were not adjacently loaded. **c.** The localization of SAMHD1 mutants was assessed in differentiated U937 cells by immunofluorescence using an anti-HA antibody followed by anti-isotype secondary antibody coupled to the $Alexa_{484}$ dye. Nuclei were stained with DAPI. Images from one representative experiment are shown (n = 2) that were acquired with an Axiovert 200 M inverted microscope, using a Plan Apo 63/1.4-N oil immersion objective. Scale bar = 10 µm. The average number of dots per cell was determined using the FiJi-ImageJ Software .

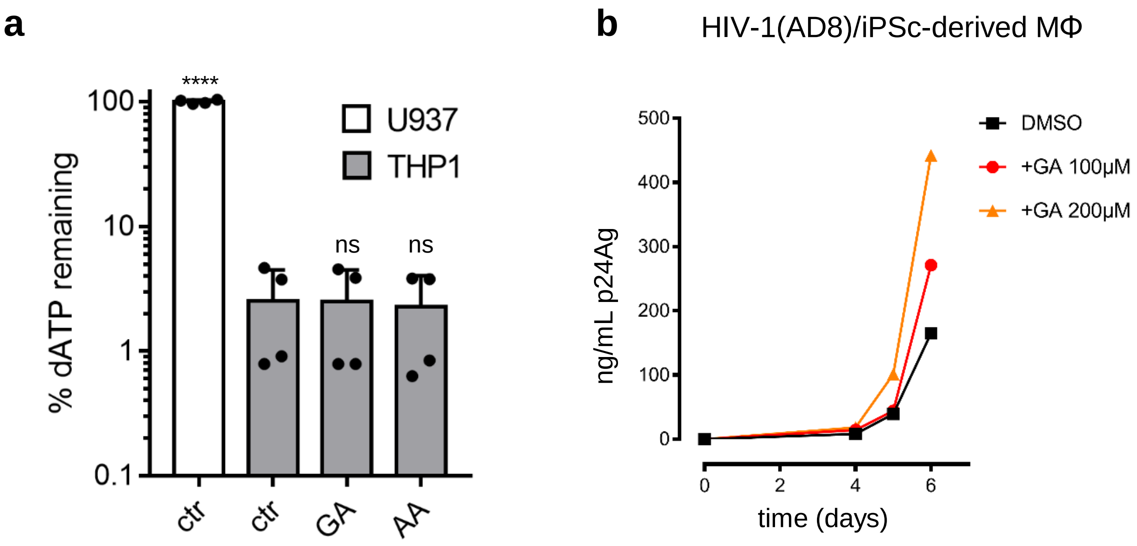

Supplementary Figure 13. Inhibition of SUMOylation enhances HIV-1 spreading in human monocyte-derived macrophages without modifying dATP levels (related to Fig. 6). a. Cellular dATP levels were quantified as in Fig. 3d. The dNTP levels of U937 were set to 100 %. Bars show the mean ± SD (n=4). Statistical significance was assessed by one-way ANOVA test. ****: p<0.0001. ns: not significant. **b.** Viral replication kinetics in macrophages derived from induced-pluripotent stem cells (iPSc) pretreated with ginkgolic acid (GA, 2h) and then challenged with the AD8 HIV-1 strain (10 ng/mL p24Ag). Viral replication was analyzed as in Fig. 6C.

Supplementary Table 1. Compilation of SAMHD1 residues identified as potential SUMOylation target sites in large-scale proteomic studies.

	Ref #	1	2	3	4	5	6	7	8	9	10	11	12	13	14	15	16	17	18	19	20	21	22	24	25	26	27	28
**Position (Human SAMHD1)**		**11**	**43**	**66**	**148**	**288**	**294**	**304**	**332**	**336**	**354**	**377**	**405**	**437**	**446**	**455**	**467**	**469**	**478**	**484**	**486**	**492**	**494**	**523**	**534**	**544**	**595**	**622**
**Study count**		**1**	**2**	**2**	**1**	**1**	**2**	**1**	**1**	**1**	**1**	**1**	**1**	**1**	**2**	**2**	**3**	**7**	**1**	**1**	**1**	**2**	**2**	**2**	**2**	**3**	**5**	**6**
Hendriks & Nielsen et al. (2018)	4		+				+										+	+						+	+	+	+	
Hendriks & Nielsen et al. (2017) ***Fractional protein intensity***[a]	5	0.7	0.1	0.4		0.2	4.4	0.3		0.1	0.1	0.1	0.1	0.1	0.1	0.5	1.3	61.2			0.3	0.9	4.0	0.4		0.2	7.1	17.7
Co-modification							Ub, Ac	Ub					Ac			Ub										Ub	Ph (~20%)	
Lamoliatte & Thibault et al. (2017) / MG	6			+	+										+	+	+	+		+		+	+		+	+	+	+
Lumpkin & Komives et al. (2017)	7								+									+	+								+	+
Tammsalu & Hay et al. (2014) / HS	8																	+									+	+
Hendriks & Vertegaal et al. (2014)	9																	+										+
Lamoliatte & Thibault et al. (2014) / MG	10																	+										+

[a]Contribution of a SUMO site to the overall SUMOylation of the protein.

Supplementary Table 2. List of the oligos used in this study.

Name	Mutation	Sequence	Mer	GC %	MW	Ta (°)
CMV forward primer	-	cgcaaatgggcggtaggcgtg	21	67	6552,3 Da	
HAsam_middle_F	-	aggaacaaattgtaggaccac	21	43	6472,3 Da	
Samhd1 Rev	-	aagccatacgggaagcaatagc	22	50	6786,5 Da	
p545_fw	K469R	aagggaggactatgaatctcttccaaaag	29	41	8958,9 Da	59
p545_rev		ctaatctttatttgtcctgttggctg	26	38	7924,2 Da	
p546_fw	E471Q	ggactatgaatctcttccaaaagaggttg	29	41	8940,9 Da	59
p546_rev		tgccttttaatctttatttgtcctgttgg	29	34	8836,8 Da	
p547_fw	K622R	agagatgacccaatgtgactcgacaatcaac	31	45	9506,3 Da	60
p547_rev		aaaaagctggactctgcttttggatgcttc	30	43	9212,1 Da	
p548_fw	D624N	cccaatgtgactcgacaatcaacctctggattac	34	47	10 330,8 Da	61
p548_rev		ttatctttaaaaagctggactctgcttttggatg	34	35	10 452,9 Da	
p558_fw	E597Q	atggaacgacagtacttcagtccaaaatccaac	33	42	10 083,6 Da	60
p558_rev		tgcttttttgaggtgttatgagtggggctataac	35	40	10 869,2 Da	
p559_fw	K595A	aaaggaatggaacgacagtacttcag	26	42	8061,3 Da	62
p559_rev		gcttgaggtgttatgagtggggctataac	29	48	9044,0 Da	
p595_fw	ΔC595	aacacctcaataaaaggaatggaac	25	36	7685,1 Da	61
p595_rev		atgagtggggctataacatc	20	45	6181,1 Da	
p624_fw	K595R	acacctcaaagaaaggaatggaac	24	42	7396,9 Da	59
p624_rev		tatgagtggggctataac	18	44	5578,7 Da	
p625_fw	T592E	cccactcatagaacctcaaaaaaagg	26	42	7910,2 Da	59
p625_rev		gctataacatcgccatcc	18	50	5403,6 Da	
p655_fw	T592A	cccactcatagcacctcaaaaaaag	25	44	7557,0 Da	62
p629_fw	Q594N	caaaaaggaatggaacgacagtac	24	42	7436,9 Da	63
p629_rev		ttaggtgttatgagtggggctataac	26	42	8096,3 Da	
p652_fw	SIM2m(LLDV/AADA)	gacgcaaaactgaaggctgaagattttatag	31	39	9616,4 Da	61
p652_rev		ggcggctactttgggtttagcactg	25	56	7720,1 Da	
p683_fw	T592A + K595R	gaaggaatggaacgacagtacttcag	26	46	8077,3 Da	64
p683_rev		ctttgaggtgctatgagtggggc	23	57	7166,7 Da	
p703_fw	ΔC597 SalI (to generate SUMO chimerae)	tcgacaatcaacctctgg	18	50	5443,6 Da	57
p703_rev		cctttttttgaggtgttatg	20	35	6135,1 Da	
p721_fw	ΔC600	tgacccaatgtgactcgac	19	53	5851,8 Da	63
p721_rev		gttccattcctttttttgaggtg	23	39	7017,6 Da	
p747_fw	ΔC597	aaaaggtcgactaacaacctctggattac	29	41	8878,9 Da	63
p747_rev		tttgaggtgttatgagtgg	19	42	5945,0 Da	
p704_fw	SUMO1 WT	ccatctcgagtctgaccaggaggcaaaacc	30	57	9170,0 Da	65
p704_rev		cgtgtcgactcaaccccccgtttgttcctg	30	60	9060,9 Da	
p705_fw	SUMO2 WT	ccatctcgagtccgaggagaagcccaag	28	61	8592,6 Da	64
p705_rev		cgtgtcgacctaacctcccgtctgctgct	29	62	8756,7 Da	
p713_fw	SUMO1 GG/AA	gaacaaacggcggcttgagtcgac	24	58	7411,9 Da	57
p713_rev		ctgataaacttcaatcacatc	21	33	6333,2 Da	
p714_fw	SUMO2 GG/AA	cagcagacggcggcttaggtcgacaatc	28	61	8623,6 Da	60
p714_rev		ctggaacacgtcgatggt	18	56	5539,7 Da	

Supplementary References

1. Corpet, F. Multiple sequence alignment with hierarchical clustering. Methods 12, 8235–8251 (1984).
2. Robert, X. & Gouet, P. Deciphering key features in protein structures with the new ENDscript server. Nucleic Acids Res. 42, W320-4 (2014).
3. Beauclair, G. et al. JASSA: A comprehensive tool for prediction of SUMOylation sites and SIMs. Bioinformatics 31, 3483–3491 (2015).
4. Hendriks, I. A. et al. Site-specific characterization of endogenous SUMOylation across species and organs. Nat. Commun. 9, 1–17 (2018).
5. Hendriks, I. A. et al. Site-specific mapping of the human SUMO proteome reveals co-modification with phosphorylation. Nat. Struct. Mol. Biol. 24, 325–336 (2017).
6. Lamoliatte, F., McManus, F. P., Maarifi, G., Chelbi-Alix, M. K. & Thibault, P. Uncovering the SUMOylation and ubiquitylation crosstalk in human cells using sequential peptide immunopurification. Nat. Commun. 8, 14109 (2017).
7. Lumpkin, R. J. et al. Site-specific identification and quantitation of endogenous SUMO modifications under native conditions. Nat. Commun. 8, (2017).
8. Tammsalu, T. et al. Proteome-wide identification of SUMO2 modification sites. Sci. Signal. 7, 1–11 (2014).
9. Hendriks, I. A. et al. Uncovering global SUMOylation signaling networks in a site-specific manner. Nat. Struct. Mol. Biol. 24, 927–36 (2014).
10. Lamoliatte, F. et al. Large-scale analysis of lysine SUMOylation by SUMO remnant immunoaffinity profiling. Nat. Commun. 5, (2014).
