# Supplementary material for: SUMOylation of SAMHD1 at Lysine 595 is required for HIV-1 restriction in non-cycling cells
Source: Nat Commun. 2021 Jul 28;12:4582. doi: 10.1038/s41467-021-24802-5 (PMC8319325; doi:10.1038/s41467-021-24802-5)
Supplement: Supplementary file 3 — Reporting Summary [file 41467_2021_24802_MOESM3_ESM.pdf]

nature research

Corresponding author(s): Alessia Zamborlini
Last updated by author(s): May 30, 2021

Reporting Summary

Nature Research wishes to improve the reproducibility of the work that we publish. This form provides structure for consistency and transparency in reporting. For further information on Nature Research policies, see our Editorial Policies and the Editorial Policy Checklist.

Statistics

For all statistical analyses, confirm that the following items are present in the figure legend, table legend, main text, or Methods section.

n/a	Confirmed	
☐	☒	The exact sample size (n) for each experimental group/condition, given as a discrete number and unit of measurement
☐	☒	A statement on whether measurements were taken from distinct samples or whether the same sample was measured repeatedly
☐	☒	The statistical test(s) used AND whether they are one- or two-sided<br>*Only common tests should be described solely by name; describe more complex techniques in the Methods section.*
☒	☐	A description of all covariates tested
☐	☒	A description of any assumptions or corrections, such as tests of normality and adjustment for multiple comparisons
☐	☒	A full description of the statistical parameters including central tendency (e.g. means) or other basic estimates (e.g. regression coefficient) AND variation (e.g. standard deviation) or associated estimates of uncertainty (e.g. confidence intervals)
☐	☒	For null hypothesis testing, the test statistic (e.g. F, t, r) with confidence intervals, effect sizes, degrees of freedom and P value noted<br>*Give P values as exact values whenever suitable.*
☒	☐	For Bayesian analysis, information on the choice of priors and Markov chain Monte Carlo settings
☒	☐	For hierarchical and complex designs, identification of the appropriate level for tests and full reporting of outcomes
☒	☐	Estimates of effect sizes (e.g. Cohen's d, Pearson's r), indicating how they were calculated

Our web collection on statistics for biologists contains articles on many of the points above.

Software and code

Policy information about availability of computer code

Data collection	No software was used
Data analysis	Graph Prism 7.0<br>Flowjo 10.5.2<br>Fiji (https://imagej.net/ImageJ)<br>icy (http://icy.bioimageanalysis.org/)<br>jassa (http://jassa.fr/)<br>Multalin (http://multalin.toulouse.inra.fr/multalin/)<br>ESPRIT3 (http://espript.ibcp.fr/ESPript/ESPript/)<br>PyMOL v1.7<br>BD FACSDiva 9.0

For manuscripts utilizing custom algorithms or software that are central to the research but not yet described in published literature, software must be made available to editors and reviewers. We strongly encourage code deposition in a community repository (e.g. GitHub). See the Nature Research guidelines for submitting code & software for further information.

Data

Policy information about availability of data

All manuscripts must include a data availability statement. This statement should provide the following information, where applicable:

- Accession codes, unique identifiers, or web links for publicly available datasets
- A list of figures that have associated raw data
- A description of any restrictions on data availability

Original data supporting the findings of this study are available in the Source Data file or from the corresponding authors upon reasonable request. The crystal structure of human SAMHD1 was already available in the Protein Data Bank under accession code 4BZC (DOI: 10.1038/NSMB.2692).

Field-specific reporting

Please select the one below that is the best fit for your research. If you are not sure, read the appropriate sections before making your selection.

[x] Life sciences [ ] Behavioural & social sciences [ ] Ecological, evolutionary & environmental sciences

For a reference copy of the document with all sections, see nature.com/documents/nr-reporting-summary-flat.pdf

Life sciences study design

All studies must disclose on these points even when the disclosure is negative.

Sample size	No statistical methods were used to pre-determine sample size. Sample size was chosen based on previous experience and standards in the field. For flow cytometry, we analyzed 10,000 cells / sample. Each infection experiment was performed in 3 to 4 technical replicates.
Data exclusions	No data were excluded.
Replication	All experiments presented were reliably reproduced at least twice. For experiments based on U937 cell lines, at least two independent transductions were performed and analyzed.
Randomization	No formal randomization method was used. In experiment using various treatments, cells were randomly attributed to control or experimental group (Fig 1- panels A, D, E, F-, Fig 4C, Fig 5, Supp FIG 2, Supp Fig 13). For the other experiments, allocation into experimental group was irrelevant.
Blinding	Investigators were blinded to group allocation during data analysis for dNTP measurements and PLA tests. All the other experiments were not blinded because they were not deemed to be influenced by human interpretation.

Reporting for specific materials, systems and methods

We require information from authors about some types of materials, experimental systems and methods used in many studies. Here, indicate whether each material, system or method listed is relevant to your study. If you are not sure if a list item applies to your research, read the appropriate section before selecting a response.

Materials & experimental systems

n/a	Involved in the study	
[ ]	[x]	Antibodies
[ ]	[x]	Eukaryotic cell lines
[x]	[ ]	Palaeontology and archaeology
[x]	[ ]	Animals and other organisms
[x]	[ ]	Human research participants
[x]	[ ]	Clinical data
[x]	[ ]	Dual use research of concern

Methods

n/a	Involved in the study	
[x]	[ ]	ChIP-seq
[ ]	[x]	Flow cytometry
[x]	[ ]	MRI-based neuroimaging

Antibodies

Antibodies used	sheep anti-SUMO1 (Enzo Life Science, BML-PW0505, currently discontinued) used at 1:1000 (WB)<br>rabbit anti-SUMO1 (Abcam, Ab32058) used at 1:1000 (PLA)<br>rabbit anti-SUMO2/3 (Abcam, Ab3742) used at 1:1000 (WB), 1:3500 (PLA)<br>mouse anti-SUMO2/3 (Abcam, Ab81371) used at 1:4000-1:8000 (PLA)<br>mouse anti-SAMHD1 (Abcam, Ab67820) used at 1:1000 (WB), 1:5000 (IP), 1: 2500 (PLA)<br>rabbit anti-SAMHD1 (Abcam, Ab177462) used at 1:4000-1:8000 (PLA)<br>rabbit anti-pT592-SAMHD1 (Cell Signaling, #89930) used at 1:1000 (WB)

	rabbit anti-actin (Sigma-Aldrich, AA20-33) used at 1:2500 (WB)<br>anti-HA HRP (Roche, Clone 3F10) used at 1:2500 (WB)<br>anti-mouse Alexa-Fluor488, Goat (INVITROGEN, 1890503) used at 1:800 (IF)<br>anti-mouse Alexa-Fluor595, Goat (INVITROGEN, 1830459) used at 1:800 (IF)
Validation	The mouse anti-HA and mouse anti-SAMHD1 antibodies recognize a band of the appropriate size (~70 kDa) in SAMHD1-positive but not SAMHD1-negative cells by IP assay and/or Western blotting (i.e. Fig 1B and 1C for anti-HA, Fig 1D for anti-SAMHD1) and immunofluorescence/Proximity Ligation Assay (Fig. 1E for anti-SAMHD1; Supp Fig 6A for anti-HA). The rabbit anti-pT592-SAMHD1 was validated by Western blotting using SAMHD1 mutants where T592 was mutated to prevent phosphorylation (Fig. 5A). We show that the sheep anti-SUMO1 antibody (Enzo Life Science, BML-PW0505, discontinued) and rabbit anti-SUMO2/3 (Abcam, Ab3742) recognize specifically the cognate SUMO isoforms (expected size ~15 kDa) ectopically expressed by transfection in 293T cells (Fig. 1A). The rabbit anti-SUMO1 antibody (Abcam, Ab32058) has been validated by the manufacturer on human HAP1-KO cells (see website). All the commercial antibodies used in this study have been validated by the manufacturer for the detection of the human protein in the following applications: WB by the detection of a band of the expected size or immunofluorescence by the visualization of the known subcellular localization of the targeted protein.

Eukaryotic cell lines

Policy information about cell lines

Cell line source(s)	Human Embryonick kidney (HEK) 293T cells available in-house were provided by E. Payen (INSERM UMR 1184, Fontenay aux Roses, France) .<br>U937 were purchased from ATCC (ATCC® CRL-1593.2™)<br>THP1 were provided by A. Puissant (INSERM U944, Paris, France)<br>SAMHD1-KO THP1 were provided by F. Diaz-Griffero (Albert Einstein School of Medicine, Bronx, NYC, USA)
Authentication	None of the cell lines used has been authenticated by Standards of Cell Line Authentification (Almeida et al, 2016). However, THP1 and U937 were controlled by the presence and absence of SAMHD1, respectively, and relative susceptibility to infection by HIV-1, HIV-2 and HIV-2deltaVpx. HEK293T were identified based on morphological parameters and being highly transfectable by Calcium phosphate precipitation technique.
Mycoplasma contamination	All cell lines were tested mycoplasma-free (Mycoplasmacheck, GATC Biotech)
Commonly misidentified lines<br>(See ICLAC register)	No misidentified cell lines were used in this study.

Flow Cytometry

Plots

Confirm that:

☒ The axis labels state the marker and fluorochrome used (e.g. CD4-FITC).

☒ The axis scales are clearly visible. Include numbers along axes only for bottom left plot of group (a 'group' is an analysis of identical markers).

☒ All plots are contour plots with outliers or pseudocolor plots.

☒ A numerical value for number of cells or percentage (with statistics) is provided.

Methodology

Sample preparation	Cells were harvested and fixed with 4% PFA.
Instrument	BD LSRFortessa, with 5 lasers (UV, violet, bleu, yellow-green, red) and18 detectors of fluorescence (+SSC FSC). Equipped with a module HTS for 96 / 384-well plates.
Software	BD FACSDiva v9.0 or FlowJo v10.5.2 softwares were used to collect or analyze flow cytometry data, respectively. Further data analysis was performed using the statistical software package Prism v7.
Cell population abundance	Flow cytometry was used for quantification purposes only (i.e. no post-sorting fractions were collected)
Gating strategy	For all experiments FSC-A/ SSC-A gates of the starting cell population were used to discriminate between viable cells and cell debris. Singlet and doublet cells were discriminated using FSC-A/ FSC-W gating. Non-infected cells were used as negative controls.

☒ Tick this box to confirm that a figure exemplifying the gating strategy is provided in the Supplementary Information.
